# Assessment of Internet Hospitals in China During the COVID-19 Pandemic: National Cross-Sectional Data Analysis Study

**DOI:** 10.2196/21825

**Published:** 2021-01-20

**Authors:** Xingyan Xu, Yingying Cai, Siying Wu, Jianhui Guo, Le Yang, Jieli Lan, Yi Sun, Bingbing Wang, Jieyu Wu, Tinggui Wang, Shuna Huang, Yawen Lin, Yuduan Hu, Mingjun Chen, Xuecai Gao, Xiaoxu Xie

**Affiliations:** 1 Department of Epidemiology and Health Statistics School of Public Health Fujian Medical University Fuzhou China; 2 Department of Preventive Medicine School of Public Health Fujian Medical University Fuzhou China; 3 Department of Clinical Research and Translation Center Office The First Affiliated Hospital of Fujian Medical University Fuzhou China

**Keywords:** internet hospital, COVID-19, prevention, control, health care, China, cross-sectional, digital health, accessibility

## Abstract

**Background:**

Internet hospitals in China are being rapidly developed as an innovative approach to providing health services. The ongoing COVID-19 pandemic has triggered the development of internet hospitals that promote outpatient service delivery to the public via internet technologies. To date, no studies have assessed China's internet hospitals during the COVID-19 pandemic.

**Objective:**

This study aimed to elucidate the characteristics of China's internet hospitals and assess the health service capacity of these hospitals.

**Methods:**

Data on 711 internet hospitals were collected from official websites, the WeChat (Tencent Inc) platform, smartphone apps, and the Baidu search engine until July 16, 2020.

**Results:**

As of July 16, 2020, 711 internet hospitals were developed in mainland China. More than half of these internet hospitals (421/711, 59.2%) were established during 2019 (206/711, 29%) and 2020 (215/711, 30.2%). Furthermore, about one-third (215/711, 30.2%) of internet hospitals were established at the beginning of 2020 as an emergency response to the COVID-19 epidemic. The 711 internet hospitals consisted of the following 3 types of hospitals: government-oriented (42/711, 5.91%), hospital-oriented (143/711, 20.11%), and enterprise-oriented internet hospitals (526/711, 73.98%). The vast majority of internet hospitals were traditional hospitals (526/711, 74%). Nearly 46.1% (221/711) of internet hospitals requested doctors to provide health services at a specific web clinic. Most patients (224/639, 35.1%) accessed outpatient services via WeChat. Internet hospitals’ consulting methods included SMS text messaging consultations involving the use of graphics (552/570, 96.8%), video consultations (248/570, 43.5%), and telephone consultations (238/570, 41.8%). The median number of available web-based doctors was 43, and the median consultation fees of fever clinics and other outpatient clinics were ¥0 (US $0) per consultation and ¥6 (US $0.93) per consultation, respectively. Internet hospitals have provided various services during the COVID-19 pandemic, including medical prescription, drug delivery, and medical insurance services.

**Conclusions:**

The dramatic increase of internet hospitals in China has played an important role in the prevention and control of COVID-19. Internet hospitals provide different and convenient medical services for people in need.

## Introduction

The outbreak of the novel COVID-19 disease was first reported in January 2020, and the disease has been spreading throughout the entire globe without any indication of stopping any time soon [1,2]. This epidemic has constituted a public health emergency of pandemic proportions [3], which has created major dilemmas in all areas of health care. Such dilemmas include a lack of hospital resources, the suspension of outpatient services, inconvenience in seeking medical treatment due to social isolation, and lockdown. In addition, the human-to-human transmission of the SARS-CoV-2 virus has become the primary transmission route of COVID-19 [4].

Strict prevention measures and effective therapeutics are urgently needed for the effective control of the COVID-19 pandemic. Such measures include the promulgation of Chinese government decrees that require domestic internet hospitals to vigorously develop telemedicine services [5]. Currently, internet hospitals have been developed as a new approach for the provision of health services, including outpatient services delivered through the internet or various digital technologies [6,7]. Internet hospitals surmount geographical obstacles and time-related barriers, thereby making it easy for Chinese patients to rapidly seek doctor services [8]. Additionally, internet hospitals reduce the chance of nosocomial cross infection by potentially keeping patients away from hospitals. This protects both clinicians and other patients, which is a practice that is welcomed by most Chinese people [9]. Internet hospitals have proliferated as Chinese people continue to seek health services and psychological assistance amid the COVID-19 pandemic.

China’s new internet hospitals, which were established during the epidemic, have yet to be systematically and exhaustively assessed. In this study, we aimed to provide an overview of internet hospitals in China (ie, as of July 2020) through a national cross-sectional study that describes the characteristics of these hospitals. We also aimed to evaluate the health service capacity of internet hospitals during the COVID-19 epidemic.

## Methods

### Internet Hospital Eligibility

Data were collected by conducting a search on national websites, including the popular Baidu search engine and the official websites of the National Health Commission of the People’s Republic of China and each provincial health commission. The search was conducted by using specific Chinese search terms, such as those for “Internet hospitals,” “Internet health,” “Internet medicine,” “mobile medicine,” “mobile health,” “Telehealth,” “digital medicine,” “digital health,” “Web hospitals,” and “Cloud hospitals.” All officially registered internet hospitals were eligible for inclusion. Data from October 10, 2006 to July 15, 2020 were collected. Additionally, we used a public WeChat (Tencent Inc) account and app stores (ie, the Apple App Store, Android App Store, Huawei App Store, Xiaomi App Store, and 360 App Store) to find medical care platforms and mobile health apps that were affiliated with internet hospitals. The study region included all of mainland China, except Hong Kong, Macau, Taiwan, and several islands in the South China Sea. Ultimately, we found 711 internet hospitals.

### Data Collection

We searched for information about each internet hospital by using official websites, the WeChat platform (ie, one of the largest mobile social network apps in China, with more than 1 billion monthly active users), and smartphone apps (ie, if available). Collected data were recorded on a digital archive via Microsoft Excel. Information on internet hospital features included construction date, location, the identity of investors, hospital characteristics, and the role that the hospital played in combating COVID-19.

We assessed the consultation characteristics of internet hospitals, including access method (ie, website, app, WeChat, and other); consulting method (ie, video consultations, telephone consultations, and SMS text messaging consultations involving the use of both text and graphics); medical insurance, prescription, and drug delivery service provision; doctor source (ie, local hospital, medical union, district, and nationwide doctors); service time (ie, any time or specific times); the number of available web-based doctors who could deliver instant medical services; and the availability of outpatient departments.

In this study, the roles played by internet hospitals referred to whether the hospital provided COVID-19–related inquiries regarding fever clinic consultations, psychological counseling, the provision of COVID-19–related information, and whether there was an overseas version of an internet hospital that could help in the fight against COVID-19. When collecting information on fever clinics, we focused on how consultations were conducted (ie, manual, artificial intelligence, or both manual and artificial intelligence) and the fee per consultation. Additionally, we collected information on internet hospital–related policies and regulations that were released by the government, management agencies, medical institutions, and health care industry associations. This helped us to understand the actions taken by the health community in China during the COVID-19 outbreak and assess the role of internet hospitals in combating COVID-19. At least 2 researchers extracted data for each internet hospital independently. Any objections to the data were resolved via discussion.

### Data Analysis and Visualization

Data management and analysis were performed with Microsoft Excel 2017. Geographical and time distributions were drawn with R 4.0.2. Count data were expressed as number (percentage) and presented on bar charts, Venn diagrams, mosaic plots, Nightingale rose diagrams, and statistical maps. Skewed continuous data were expressed as median (interquartile range) and presented on Violin plots.

## Results

### Internet Hospital Characteristics

**Figure 1:**
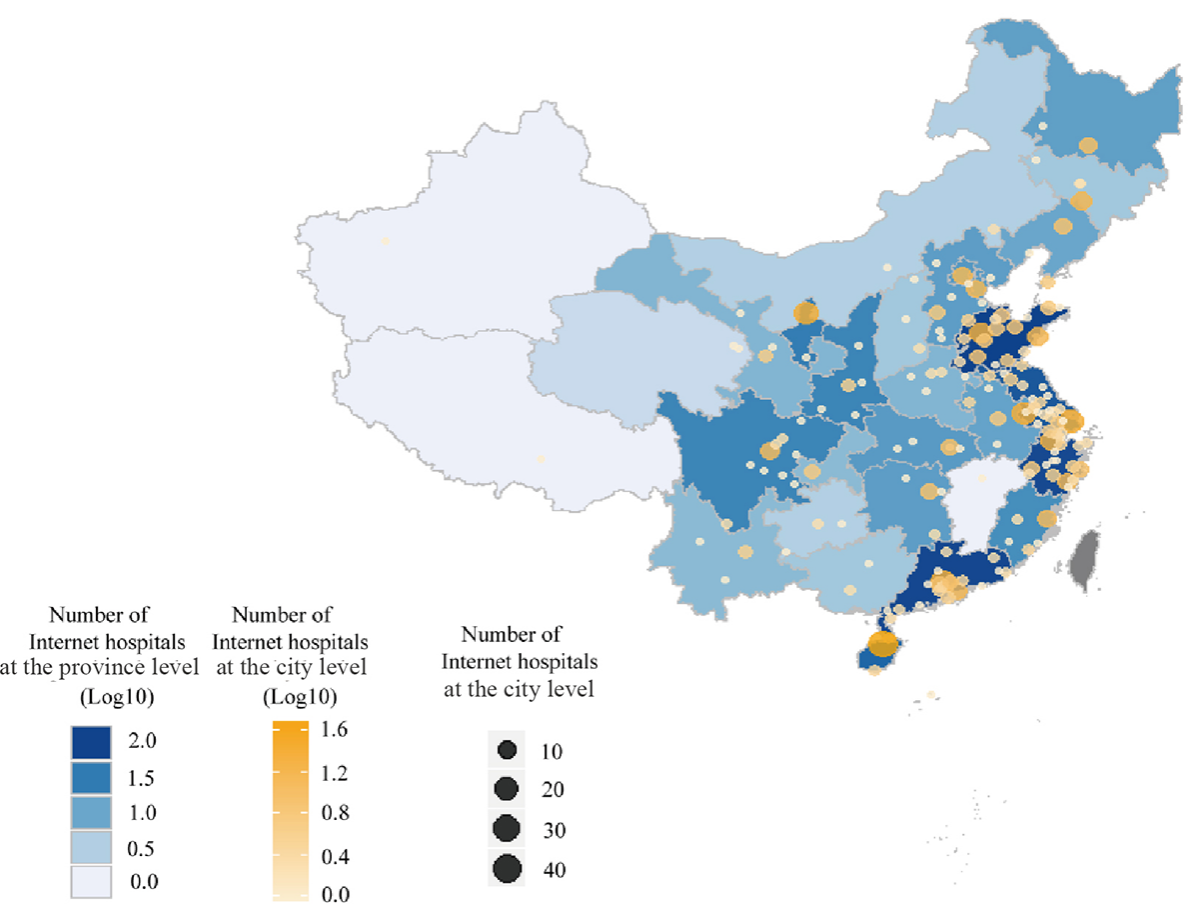
Spatial distributions of internet hospitals in China. The different shades of blue denote the total number of internet hospitals in each province. The color (ie, the different shades of yellow) and size of the circles indicate the number of internet hospitals in each city.

As of July 2020, data on 711 internet hospitals were collected. Generally, internet hospitals were mainly distributed in the east and southern coastal provinces (Figure 1). This distribution was closely related to the foundation and early development of internet hospitals in these areas [10]. However, internet hospitals were not limited to these first-to-try areas. At the time of this study, every province had at least 1 internet hospital. This reflects the trend of gradual development from coastal cities to inland regions (ie, the gradual spread of internet hospitals from the point of origin to mainland China).

**Figure 2:**
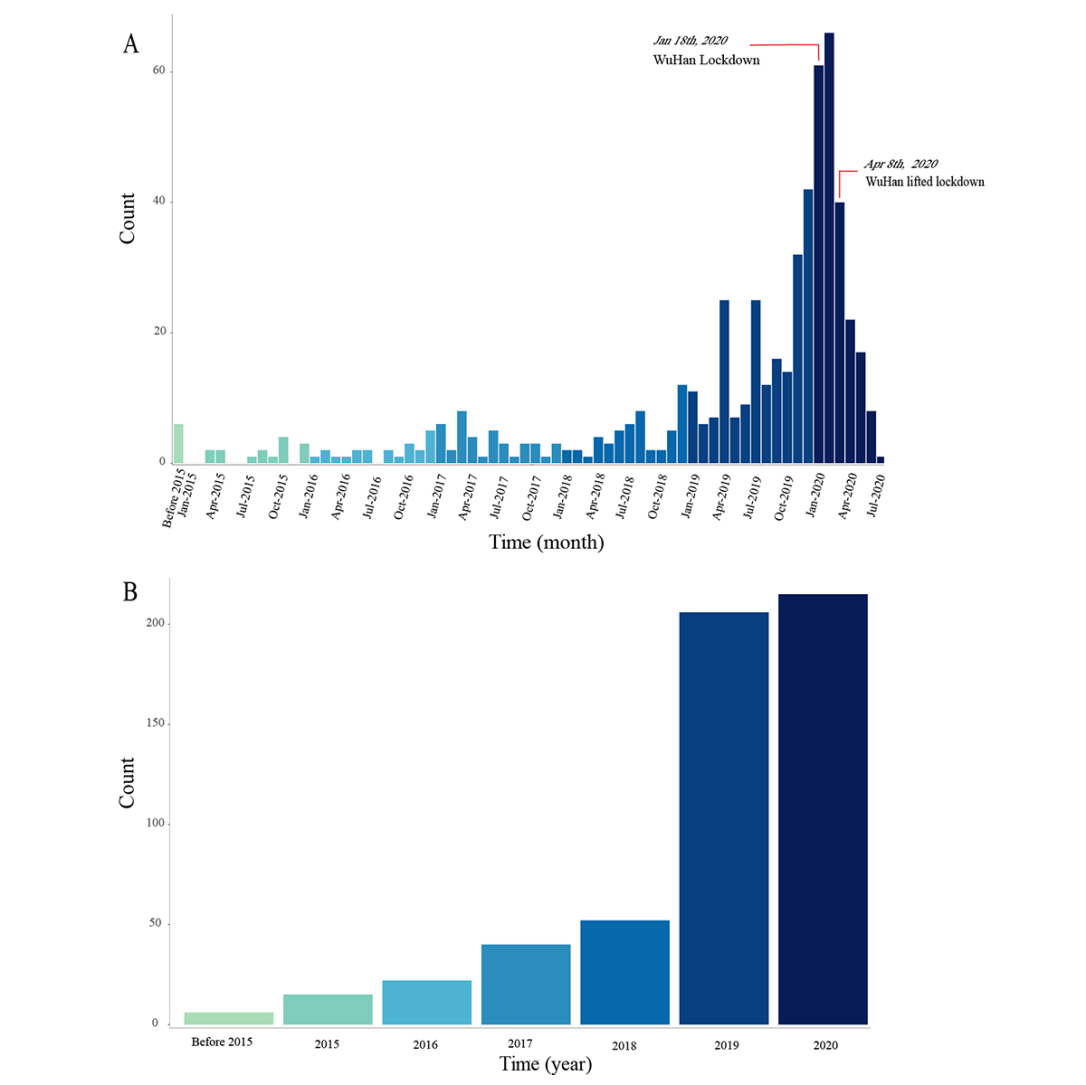
The construction dates of internet hospitals in China. (A) The construction dates of internet hospitals in China by month. (B) The construction dates of internet hospital in China by year. The following important policies were issued during the COVID-19 epidemic in China: (1) Notice on internet diagnosis and treatment consultation services for COVID-19 prevention and control (February 6, 2020), (2) Notice on strengthening internet diagnosis and treatment consulting services for COVID-19 prevention and control (February 8, 2020), (3) Notice on the national telemedicine and internet medical center for the national remote consultation of critically ill patients with COVID-19 (February 21, 2020), (4) Notice on further promoting the development and standardized management of internet medical services (April 18, 2020), and (5) Notice on the technical specifications and financial management of the "Internet+ medical service" project of public medical institutions (May 8, 2020).

In recent years, internet hospitals have been rapidly developed. Of the 711 internet hospitals found in this study, 215 (30.1%) were established in 2020 (ie, after the beginning of the COVID-19 outbreak) and 5 (0.7%) were established before 2015 and until July 2020. Moreover, 2.1% (15/711), 3.1% (22/711), 5.6% (40/711), 7.3% (52/711), 29% (206/711) and 30.2% (215/711) of internet hospitals were established in 2015, 2016, 2017, 2018, 2019, and 2020, respectively (Figure 2; Table S2 in Multimedia Appendix 1). Notably, we could not identify the date of foundation for 21.9% (156/711) of internet hospitals. We concluded that there has been a growing trend for the establishment of internet hospitals up to July 2020. Additionally, the government has issued a series of policies that were conducive to the role of internet hospitals in combating COVID-19 (Figure 2).

**Figure 3:**
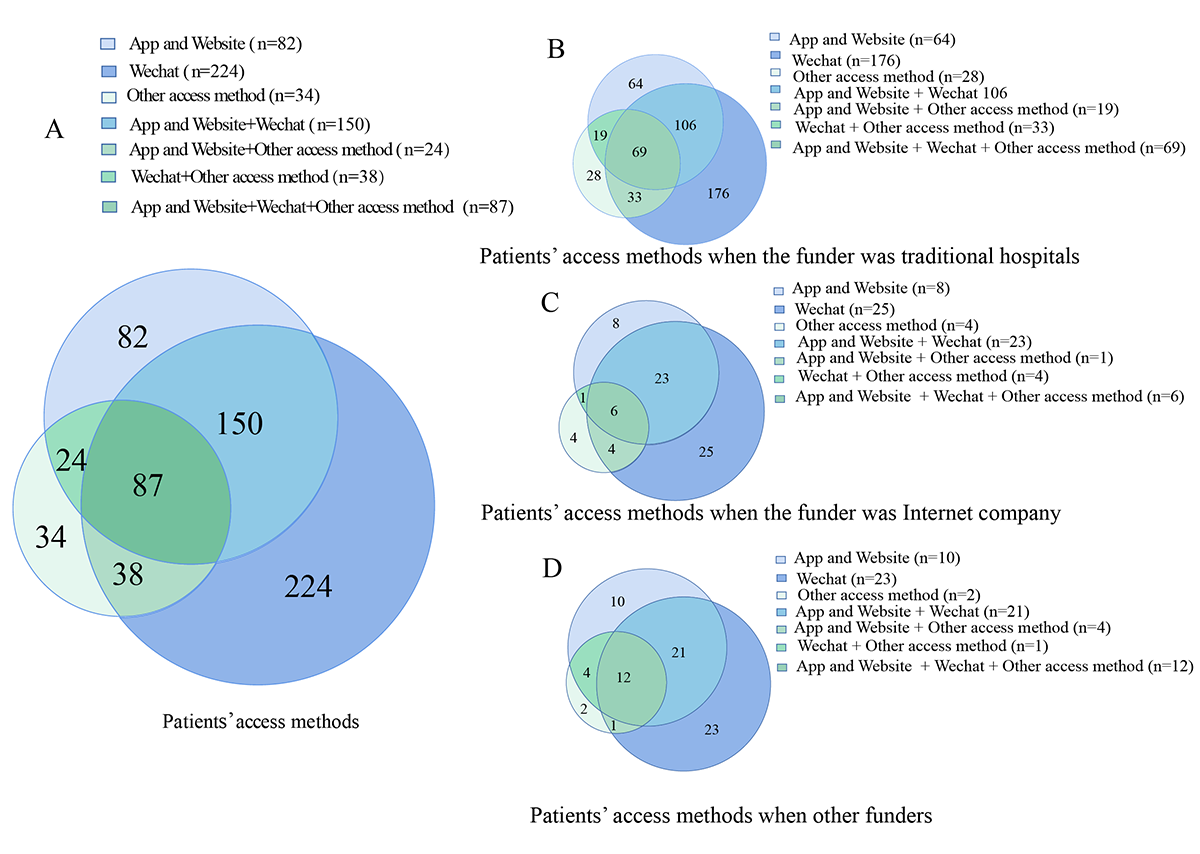
Methods for accessing internet hospitals. (A) Patients’ access methods. (B) Patients’ access methods when the funder was a traditional hospital. (C) Patients’ access methods when the funder was an internet company. (D) Patients’ access methods when the funder was another type of company.

In terms of patients’ (N=639) methods for accessing internet hospitals, 13.6% (87/639) of patients accessed internet hospitals by using apps, websites, WeChat, and other access methods simultaneously. Of note, 12.8% (82/639) of patients visited internet hospitals by exclusively using apps and websites, 35.1% (224/639) visited internet hospitals by exclusively using WeChat, and 5.3% (34/639) visited internet hospitals by using other access methods. Additionally, regardless of who funded the internet hospitals, the most widely used service that patients used to access outpatient services was WeChat, followed by apps or websites, and other access methods (Figure 3).

**Figure 4:**
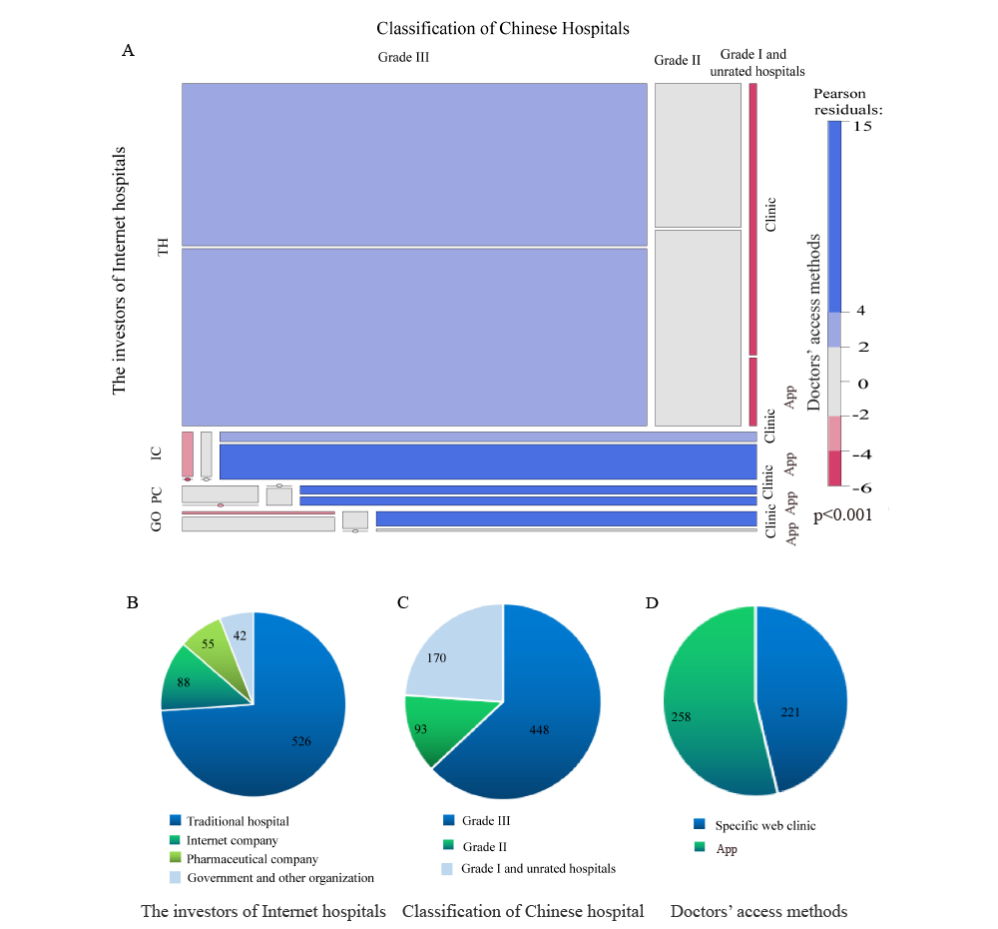
Characteristics of internet hospitals. (A) A mosaic plot with areas that show the proportion of doctors’ web-based methods for accessing internet hospitals in China, stratified by the classification of Chinese hospitals and the structure of the main investment. The investors of internet hospitals include THs, ICs, PCs, and GOs. The classification of Chinese hospitals were as follows: Grade III, Grade II, and Grade I and unrated hospitals. Doctors provided medical services through a specific web clinic or app. (B) The investors of internet hospitals. (C) Classification of Chinese hospitals. (D) Doctors’ access methods. GO: government and other organization; IC: internet company; PC: pharmaceutical company; TH: traditional hospital.

The vast majority of internet hospitals were traditional hospitals (526/711, 74%). This finding shows that traditional hospitals were more capable of integrating internet hospital services into their infrastructure than other types of hospitals. Grade III hospitals, which have a large number of outpatients, superb medical technology, diverse and complex service types, and various business process requirements, accounted for a large proportion (448/711, 62%) of internet hospitals. Furthermore, government departments, such as health administrations, also supported the construction of internet hospitals. In terms of the consultation characteristics of the 711 internet hospitals, 221 (46.14%) hospitals asked doctors to provide medical services at a specific web clinic, whereas 258 (53.86%) adopted the use of apps for health service delivery (Figure 4).

### Medical Service Characteristics

**Figure 5:**
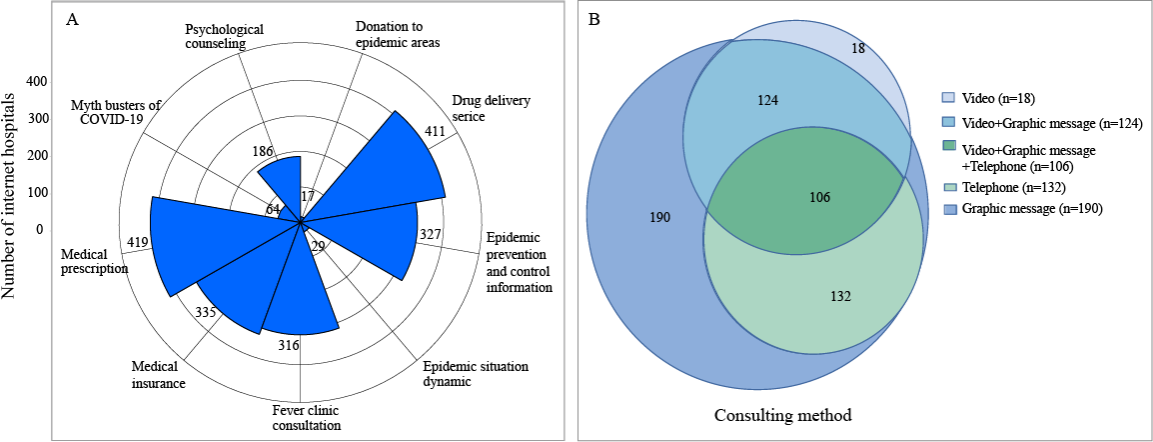
Medical service characteristics of China’s internet hospitals. (A) The roles that internet hospitals played in fighting COVID-19. (B) Internet hospitals’ consulting methods.

With regard to the roles that the 711 internet hospitals played in combating COVID-19, most hospitals (419/484, 86.6%) provided medical prescriptions. Furthermore, 74.5% of hospitals (411/552) provided drug delivery services, 67.5% (335/496) provided medical insurance, 60.7% (335/496) provided epidemic prevention and control information, 60% (316/527) provided fever clinic consultations, 35.2% (186/529) provided psychological counseling, 13.3% (64/483) provided a myth busters of COVID-19 service, 20.6% (29/141) assessed epidemic situation dynamics, and 3.8% (17/443) made donations (Figure 4). Internet hospitals’ consulting methods included SMS text messaging consultations involving the use of graphics (552/570, 96.8%), video consultations (248/570, 43.5%), and telephone consultations (238/570, 41.8%). Moreover, 18.6% (106/570) of internet hospitals offered 3 methods for patient consultations (Figure 5). It should be noted that several internet hospitals were missing pertinent data. As such, these hospitals were excluded from analysis.

### Human Medical Resources

**Figure 6:**
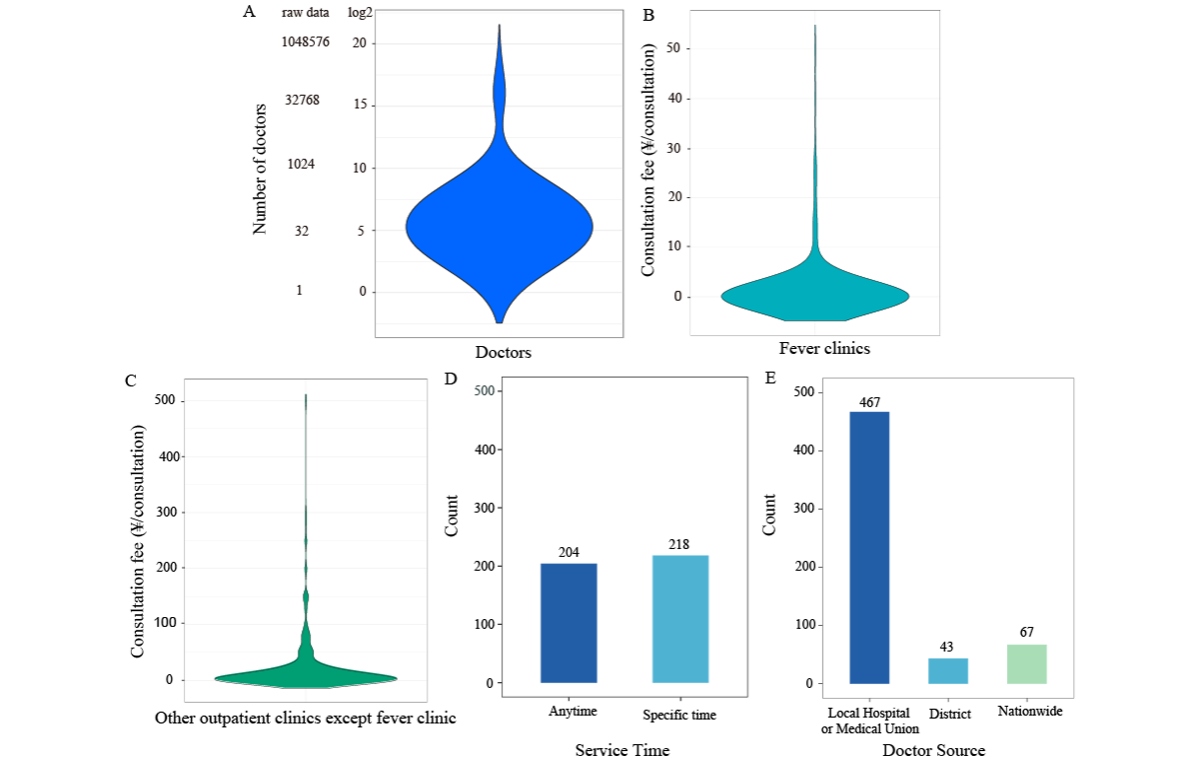
Human medical resources in China’s internet hospitals. (A) Violin plots that show the number of doctors in China’s internet hospitals. The numbers on the y-axis are logarithms to the base 2. (B, C) Violin plots displaying the consultation fees for a person who visited an internet hospital to find medical help (ie, consultation fees for fever clinics and other clinics). (D, E) Bar plots that show the distribution of doctors’ service times and doctor sources with regard to internet hospitals, respectively.

The distribution of the internet hospitals’ human medical resources in China is shown in Figure 6. The median number of available web-based doctors was 43 (IQR 3-172; maximum: 563,881). The median consultation fees of fever clinics and other outpatient clinics (ie, excluding fever clinics) were ¥0 (US $0; IQR ¥0 [US$ 0]-¥0 [US $0]) per consultation and ¥6 (US $0.93; IQR ¥0 [US $0]-¥20 [US $3.09]) per consultation, respectively (Figure 6, Table 1). About half of the hospitals (204/422, 48.3%) provided services at any time. Furthermore, 80.9% (467/577) of doctors were from local hospitals and medical unions (Figure 6).

**Table 1:** Number of doctors and consultation fees for internet hospitals in China.

Variable	Minimum	5th percentile	25th percentile	50th percentile	75th percentile	95th percentile	Maximum
Number of available web-based doctors, n	1	3	11	43	172	1496.4	563881
Consultation fee of fever clinics, ¥^a^/consultation	0	0	0	0	0	8.9	50
Consultation fee of other outpatient clinics (ie, excluding fever clinics), ¥/consultation	0	0	0	6	20	120	500

^a^A currency exchange rate of ¥1=US $0.15 is applicable.

**Figure 7:**
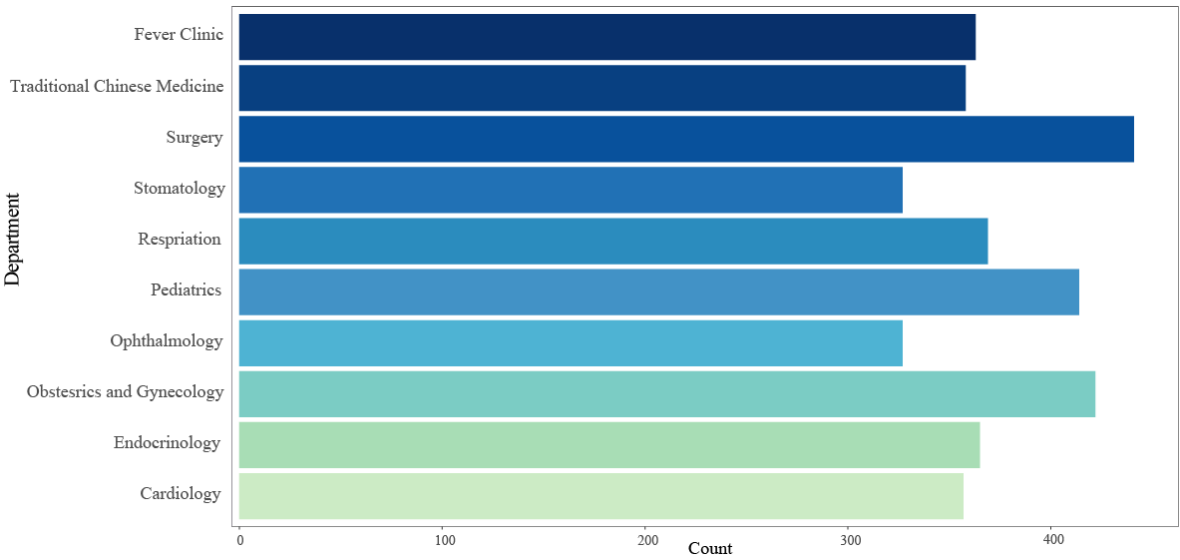
A bar plot that depicts the number of available departments in internet hospitals.

Multiple internet hospitals had different departments for patients; the department of surgery (441/711, 62%) was the most common. In contrast, the number of stomatology (327/711, 46%) and ophthalmology (327/711, 46%) departments was relatively small. Of the 711 internet hospitals, 363 (51.1%) introduced fever clinics in response to the COVID-19 pandemic (Figure 7).

### Relation to COVID-19

**Figure 8:**
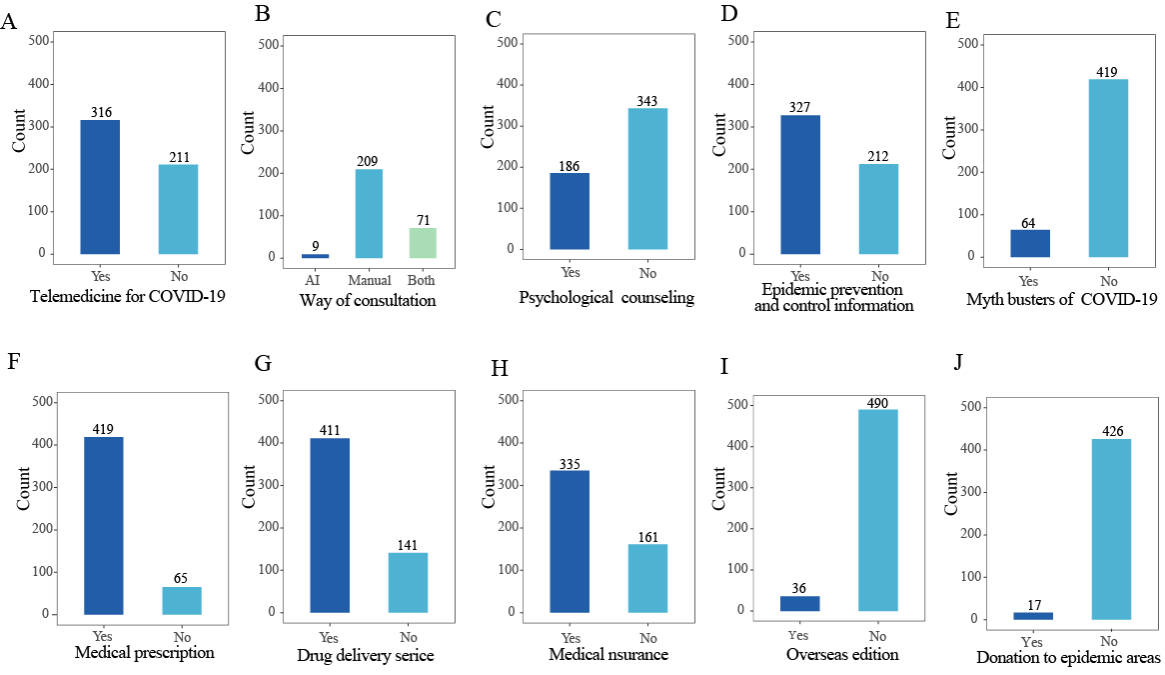
(A-J) Bar plots that depict the roles of internet hospitals during the COVID-19 epidemic. AI: artificial intelligence.

With regard to the internet hospitals with available data, 60% (316/527) of internet hospitals provided telemedicine services for managing COVID-19. Internet hospitals mainly conducted manual consultations (209/289, 72.3%). In addition, 35.2% (186/529), 60.7% (327/539), 13.3% (64/483), and 3.8% (17/443) of internet hospitals provided psychological counseling, epidemic prevention and control information, myth busters services, and donations, respectively, in response to COVID-19. Furthermore, a number of internet hospitals supported medical prescription (419/484, 86.6%), drug delivery (411/552, 74.5%), and medical insurance (335/496, 67.5%) services to further enhance COVID-19 prevention and control (Figure 8).

## Discussion

### Principal Findings

This national cross-sectional study outlines the status of the internet hospitals established in China by July 2020. There has been a rising trend in the emergence of internet hospitals in China. This trend peaked in December 2019 after the China National Healthcare Security Administration promulgated the Guidance of the State Medical Security Bureau on improving the Internet+ medical service price and medical insurance payment policy [11]. Furthermore, the number of internet hospitals peaked in February 2020. This may have been the period with the largest number of internet hospitals established in a single month due to the epidemic.

Due to inequalities in medical resources, medical technology, and informatization level across the country, the status of internet hospitals in China varies greatly. Overall, the eastern and southern coastal provinces have the highest distribution of major internet hospitals. This distribution is closely related to the foundation and early development of internet hospitals in these regions [10]. However, internet hospitals are no longer limited to these areas. Jiangxi, Jilin, and other areas established the first batch of internet hospitals in 2020. This reflects the trend of gradual development from coastal cities to inland areas.

The vast majority of internet hospitals have been constructed by Grade III hospitals, which have a large number of patients, superb medical technology, and diverse and complex health services. The independent construction of an internet hospital system can meet the needs of individualized development. Moreover, the health administration department has been promoting the construction of internet hospitals. A concern however is that in most provinces, traditional hospitals make up a large proportion of internet hospitals; only Ningxia and Hainan are dominated by corporate-led internet hospitals.

The catalytic effect of the COVID-19 pandemic on the internet hospital industry has been obvious since 2020; the pandemic has resulted in major development opportunities for internet hospitals. Due to the need for epidemic prevention and control, the National Health Commission, the National Medical Insurance Bureau, and other health departments have intensively introduced a series of policies [12-16] to vigorously promote the development of internet hospitals. During the epidemic, internet hospitals have launched web-based fever clinics; chronic disease follow-up consultations, prescriptions, and drug delivery services; and several medical insurance reimbursement programs, and these have been continuously and urgently approved. Web-based medical treatment has promoted the public awareness of internet hospitals. Additionally, the epidemic has prompted people to develop a habit of using internet hospitals, to a certain extent [17].

Internet hospitals can reduce crowd gathering in physical hospitals through a variety of methods. Web-based medical services, such as web-based education, publicity, and psychological intervention, can not only help the public master basic anti-epidemic knowledge and skills, but also reduce social panic, thereby reducing the number of unnecessary physical hospital visits and enhancing psychological resilience [18]. By integrating web-based resources with offline epidemic control measures, internet hospitals can play a greater role in epidemic prevention and control [19].

With the launch of internet hospitals, the tension regarding offline medical resources has, to a certain extent, been relieved. With regard to upper first-class hospitals, the most direct role of internet hospitals has been shifting to providing returning patients with web-based services and improving the efficiency of medical treatment. Small and medium regional hospitals with a small number of outpatients or limited construction and operation capabilities have the choice of relying on regional internet hospitals or third-party, internet-based medical platforms. This approach is meant to reduce input costs by making full use of the advantages of internet hospitals, as internet-based platforms can help doctors concentrate on becoming the core resource for serving patients. As a result, internet hospitals and internet-based platforms will achieve the advantage of complementation and achieve a win-win situation.

After several years of development, China's internet hospitals can now overcome the limitations of time and space and provide various medical services with a high degree of accessibility for all patients [20]. Moreover, interdisciplinary and transregional exchanges and cooperation can be achieved with internet hospitals, thereby improving doctors’ ability to deal with emerging diseases. The Chinese government encouraged internet hospitals to join epidemic prevention and control efforts at the beginning of the COVID-19 outbreak [11], and confirmed that internet hospitals are an important part of the joint epidemic prevention and control system [21]. To make better use of internet hospitals, more measures should be implemented, such as encouraging more doctors (ie, especially psychologists and pediatricians) to take part in web-based services, discovering the needs of the public in time to adjust response strategies, and developing more standardized consulting service guidelines. At the same time, there is a need to improve the availability of internet hospital services in a variety of ways, strengthen publicity efforts for expanding the internet hospital user base, and cooperate with community service centers and the Centers for Disease Control and Prevention to improve joint epidemic control and prevention mechanisms.

### Conclusion

This study demonstrates how the concepts of medical service provision have been redefined by internet hospitals and internet-based technologies. Internet hospitals allow for the transferring of patients to medical centers that can provide medical services and health care without requiring people to leave their homes. Internet hospitals can provide different types of medical services to people seeking medical needs; offer essential medical support to the public during the COVID-19 outbreak, thereby regulating social psychology and easing social panic; maintain proper social distancing; and promote correct medical-seeking behaviors. These advantages result in the reduction of the incidence of cross-infection in hospitals. Thus, it is evident that internet hospitals play an important role in COVID-19 prevention and control.

## Appendix

Multimedia Appendix 1 Supplementary tables.
