# Supplementary material for: Assessment of Internet Hospitals in China During the COVID-19 Pandemic: National Cross-Sectional Data Analysis Study
Source: J Med Internet Res. 2021 Jan 20;23(1):e21825. doi: 10.2196/21825 (PMC7819672; doi:10.2196/21825)
Supplement: Multimedia Appendix 1 [file jmir_v23i1e21825_app1.docx]

**Assessment of Internet Hospitals in China During the COVID-19 Pandemic: National Cross-Sectional Data Analysis Study**

**Supplement**

Table S1. The search keywords

|  | Search terms |
| --- | --- |
| In English | “Internet hospitals”, “Internet health”, “Internet medicine”, “mobile medicine”, “mobile health”, “Telehealth”, “digital medicine”, “digital health”, “Web hospitals”, and “Cloud hospitals” |
| In Chinese | “互联网医院”、“互联网健康”、“互联网医学”、“移动医学”、“移动健康”、“远程健康”、“数字医学”、“数字健康”、“网络医院”和“云医院” |

Table S2. Construction date of Internet hospital in China

|  | Jan | Feb | Mar | Apr | May | Jun | Jul | Aug | Sep | Oct | Nov | Dec | *Total* |
| --- | --- | --- | --- | --- | --- | --- | --- | --- | --- | --- | --- | --- | --- |
| Before 2015 | - | - | - | - | - | - | - | - | - | - | - | - | 5 |
| 2015 | - | - | 2 | 2 | - | - | 1 | 2 | 1 | 4 | - | 3 | 15 |
| 2016 | 1 | 2 | 1 | 1 | 2 | 2 | 0 | 2 | 1 | 3 | 2 | 5 | 22 |
| 2017 | 6 | 2 | 8 | 4 | 1 | 5 | 3 | 1 | 3 | 3 | 1 | 3 | 40 |
| 2018 | 2 | 2 | 1 | 4 | 3 | 5 | 6 | 8 | 2 | 2 | 5 | 12 | 52 |
| 2019 | 11 | 6 | 7 | 25 | 7 | 9 | 25 | 12 | 16 | 14 | 32 | 42 | 206 |
| 2020 | 61 | 66 | 40 | 22 | 17 | 8 | 1 | - | - | - | - | - | 215 |
| Unknown | - | - | - | - | - | - | - | - | - | - | - | - | 156 |
| *Total* | - | - | - | - | - | - | - | - | - | - | - | - | **711** |
